# Editorial: Women in Gastrointestinal Sciences: 2021

**DOI:** 10.3389/fphys.2022.1048724

**Published:** 2022-10-19

**Authors:** Natalie Luhtala, Kathleen E. DelGiorno

**Affiliations:** ^1^ Molecular and Cell Biology Laboratory, Salk Institute for Biological Studies, La Jolla, CA, United States; ^2^ Department of Cell and Developmental Biology, Vanderbilt University School of Medicine, Nashville, TN, United States

**Keywords:** women, gastrointestinal, spotlight, Cancer, female

In this Research Topic, we feature the accomplishments of gastrointestinal (GI) tract researchers who identify as female. Women account for less than 30% of scientists worldwide. Gender biases and stereotyping may prevent women from pursuing careers in science. Women who choose scientific careers may experience discrimination through lower pay, fewer resources, and decreased recognition and advancement as compared to their male colleagues.

The GI tract, which includes the pancreas, liver, gallbladder, intestines, and colon, consists of multiple organs that form a continuous tract from the mouth to the anus, coordinating food intake and digestion. Herein, we highlight the work of female scientists, covering multiple areas of GI research. Reviews address advancements in the diagnosis of pancreatic intraductal neoplasms (Assarzadegan et al.), detail the relevance of purinergic and adenosinergic signaling in pancreatobiliary diseases (Faraoni et al.), and examine the role of a cystine/glutamate antiporter in GI cancer (Jyotsana et al.). Clinical questions were explored in research articles analyzing how pancreas fat contributes to metabolic risk of type 2 diabetes (Sequeira et al.) and in a study probing the use of CT scans for osteoporosis screening in pancreatitis (McNabb-Baltar et al.). In two cancer research articles, immunofluorescent techniques were developed to define the emergence of enteroendocrine cells early in pancreatic tumorigenesis (Caplan et al.) and to characterize a unique tumor microenvironment in inflammation-induced colon cancer (Vega et al.). Studies of *Acinetobacter calcoaceticus* revealed its adaptation to GI stressors and its proposed influence on the gut epithelium through pro-inflammatory signaling pathways (Glover et al.).

**FIGURE 1:**
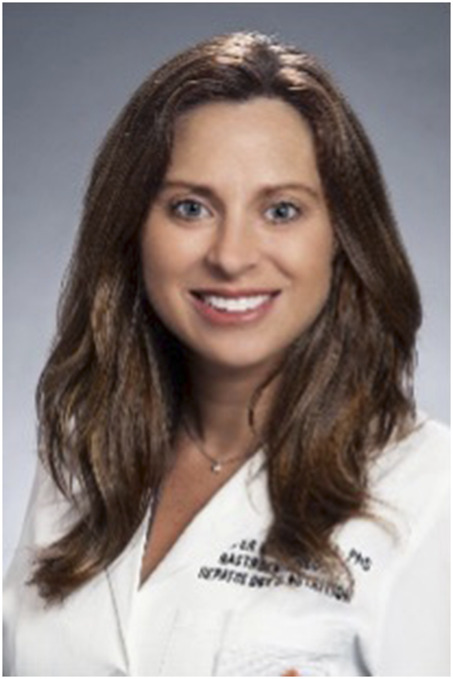
Dr. Jennifer Bailey-Lundberg.

Bailey-Lundberg (Figure 1) studies the interplay between the epithelium and immune system in chronic inflammation and cancer with a major focus on pancreatic diseases. Her lab uses genetically engineered mouse models to evaluate plasticity in acinar and ductal cells as well as how signaling from the epithelium alters the immune and stromal microenvironment. Most recently her lab has been focused on studying purinergic and adenosine signaling with therapeutic implications for immunoprevention in pancreatic cancer.

**FIGURE 2:**
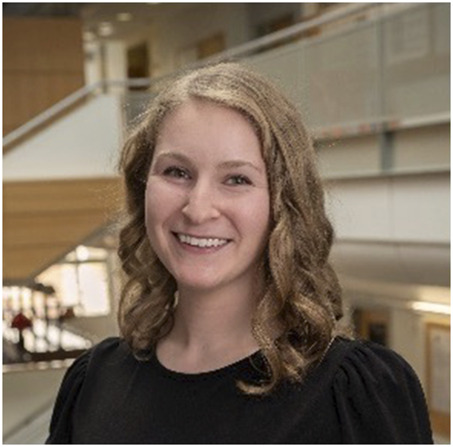
Leah Caplan.

Caplan (Figure 2) is a PhD candidate in the laboratory of Dr. Kathleen DelGiorno at Vanderbilt University. Pancreatic cancer is set to become the second leading cause of cancer-related deaths by 2030, however how it develops is still poorly understood. Acinar-to-ductal metaplasia (ADM), a process in which acinar cells transdifferentiate into ductal-like cells in response to injury, is an early event in tumorigenesis. ADM yields a heterogeneous population of cells including hormone-producing enteroendocrine cells rarely present in the normal pancreas. Leah Caplan is studying the role of enteroendocrine cells in pancreatic injury, repair, and tumorigenesis.

**FIGURE 3:**
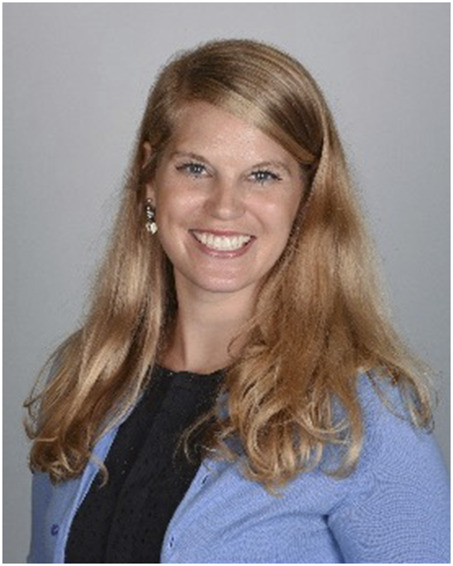
Dr. Melinda Engevik.

*Dr. Mindy (Melinda) Engevik* (Figure 3) is an Assistant Professor at the Medical University of South Carolina. Her lab focuses on the interaction between the gut microbiota and the intestinal epithelium, with an emphasis on mucus-associated microbes. Mindy complements her microbiology work with intestinal organoids, which provides a novel reductionist model of microbe-host interactions.

**FIGURE 4:**
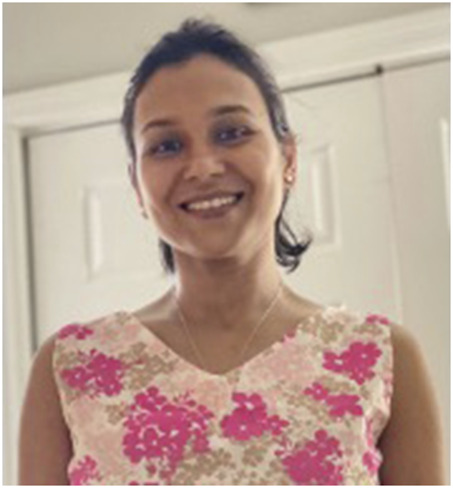
Dr. Nidhi Jyotsana.

Jyotsana (Figure 4) is a Research Assistant Professor in the DelGiorno Laboratory at Vanderbilt University. Applying her skills as a bioengineer, she is utilizing clinically advanced lipid nanoparticles as vehicles to package and deliver RNA molecules to pancreatic tissue *in vivo* in models of intraductal papillary neoplasms (IPMN) and pancreatic cancer. Dr. Jyotsana aims to understand the molecular mechanisms of IPMN and pancreatic cancer pathogenesis, to identify novel therapeutic targets, and to efficiently and safely target IPMN and pancreatic cancer. Dr. Jyotsana is a recent recipient of a Department of Defense Partnering PI Award in Pancreatic Cancer.

**FIGURE 5:**
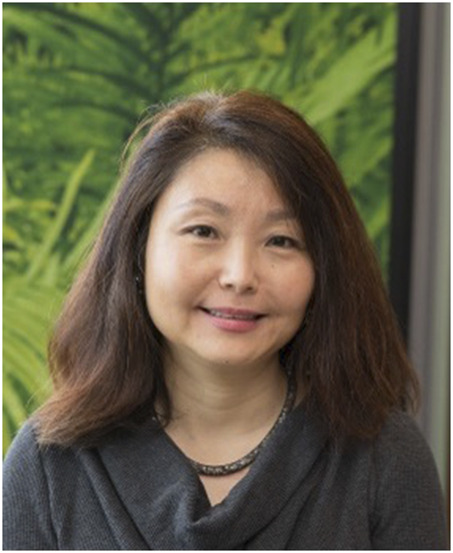
Dr. Jiaqi Shi.

Shi (Figure 5) is an Associate Professor of Pathology at the University of Michigan. She is a fellowship-trained and board-certified Gastrointestinal, Pancreas, and Hepatobiliary Pathologist and an NIH-funded physician-scientist. Her research interests are epigenetic regulation and tumor microenvironment in pancreatic cancer development and pancreas pathology. Dr. Shi has won the Benjamin Castleman Award from the United States and Canadian Academy of Pathology (USCAP) and is the recipient of an NCI Mentored Clinical Scientist Research Career Development Award (K08) and MERIT (Method to Extend Research in Time) award (R37).

**FIGURE 6:**
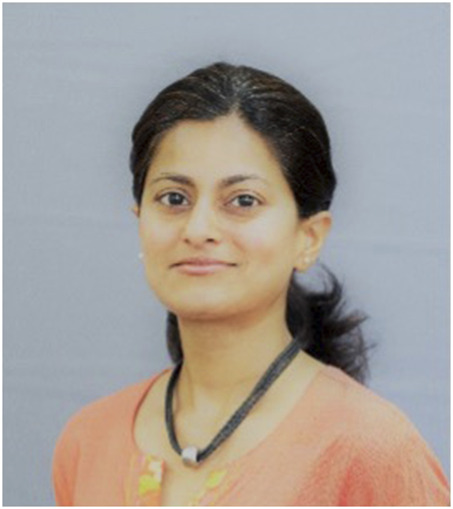
Dr. Ivana Sequeira.

Sequeira (Figure 6) explored the use of a non-invasive test of gut permeability during her doctorate and postdoctoral studies. This test was investigated as a diagnostic and used to assess how breakdown/digestion of food in the gut liberates soluble nutrients (Digesta Group at Massey University). This test was endorsed in an editorial, with funding awarded for this work (Return on Science Fund, Massey University). Recently, Dr. Sequeira led a project as a Research Fellow (University of Auckland, PANaMAH), investigating the use of MRI and Spectroscopy to quantify fat storage in key metabolic organs such as the liver and pancreas.

**FIGURE 7:**
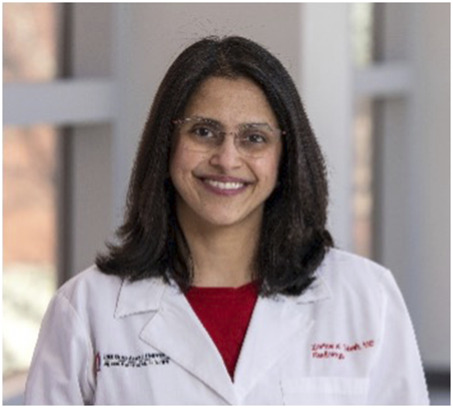
Dr. Zarine Shah.

Shah (Figure 7) is an Associate Professor of Radiology at The Ohio State University. Her career has focused on abdominal imaging, specifically in hepato-biliary and pancreatic disorders, and MRI imaging of the prostate and bladder. Dr. Shah advocates for diversity and equity, serving as Vice Chair for Diversity, Equity, and Inclusion, Associate Director of the Women in Medicine and Science committee, Chair-elect of the President and Provost’s Council on Women, and acting as interim Director of Women’s Academic Advancement. She also contributes her time by providing faculty training as part of the President and Provost’s Leadership Institute.

**FIGURE 8:**
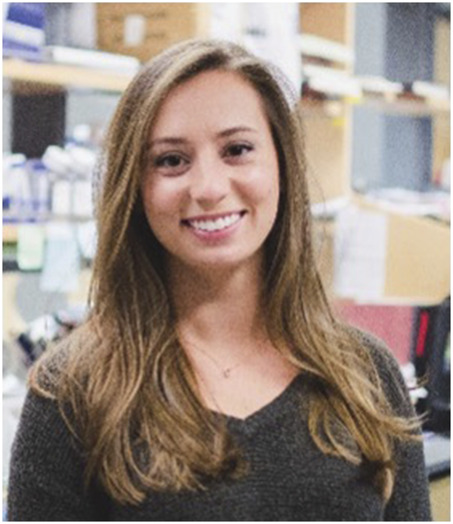
Paige Vega.

Vega (Figure 8) is a PhD candidate in Dr. Ken Lau’s laboratory within the Department of Cell and Developmental Biology and the Epithelial Biology Center at Vanderbilt University. Her work leverages both experimental and computational approaches to investigate epithelial-stromal cell-cell interactions in intestinal inflammation. In her publication featured in this Research Topic, she used single-cell RNA-sequencing, multiplex immunofluorescence imaging, and computational approaches to explore the colonic tumor microenvironment in mouse models of advanced adenoma and pre-invasive inflammation-induced cancer. Her goal is to gain the independence, additional training, and expertise required to launch her own laboratory as an independent academic investigator in biomedical research.

